# Working memory updating training reduces state repetitive negative thinking: Proof-of-concept for a novel cognitive control training

**DOI:** 10.1016/j.brat.2021.103871

**Published:** 2021-07

**Authors:** Henrietta Roberts, Mohammod Mostazir, Nicholas J. Moberly, Edward R. Watkins, Anna-Lynne Adlam

**Affiliations:** University of Exeter, UK

**Keywords:** Rumination, Working memory, Cognitive control training, Repetitive negative thinking

## Abstract

Repetitive negative thinking (RNT) is a proximal risk factor implicated in the onset and maintenance of common mental health problems such as depression and anxiety. Adolescence may be a key developmental window in which to target RNT and prevent the emergence of such disorders. Impairments in updating the contents of working memory are hypothesised to causally contribute to RNT, and some theorists have suggested these difficulties may be specific to the manipulation of negative information. The present study compared the effects of computerised adaptive working memory updating training (in which the task becomes more difficult as performance improves) to a non-adaptive control task in reducing levels of RNT. 124 healthy young people were randomised to 20 sessions of (i) working memory updating training using neutral stimuli, (ii) working memory updating training using negative stimuli, or (iii) non-adaptive working memory updating training. Adaptive working memory updating training using neutral, but not negative, stimuli resulted in significant improvements to working memory updating for negative material, as assessed using an unpractised task, and significant reductions in susceptibility to state RNT. These findings demonstrate proof-of-concept that working memory updating training has the potential to reduce susceptibility to episodes of state RNT.

## Highlights

• 124 young people participated in a novel working memory updating training.
• Training using neutral, but not negative, stimuli improved working memory updating.
• Training using neutral stimuli also reduced repetitive negative thinking.
• Working memory updating causally influences repetitive negative thought.
• Targeted working memory updating training may be a valuable preventive approach.

Repetitive negative thinking (RNT) is a hallmark feature of depression and anxiety, and an important transdiagnostic proximal risk factor for common mental health problems (Harvey, Watkins, Mansell, & Shafran, 2004; Ehring & Watkins, 2008; McEvoy, Watson, Watkins, & Nathan, 2013; Spinhoven, van Hemert, & Penninx, 2018). RNT has been robustly implicated in the onset and maintenance of depression and anxiety, as well as multiple other mental health problems (Ehring & Watkins, 2008), and understanding the mechanisms underpinning pathological RNT is increasingly recognised as an important target for research (Watkins & Roberts, 2020). RNT is defined as a “process of thinking attentively, repetitively, or frequently about one's self and one's world” (Segerstrom, Stanton, Alden, & Shortridge, 2003) and comprises recurrent thoughts that are negative in valence and difficult to control (Ehring & Watkins, 2008). The most common forms of RNT are worry and depressive rumination, and trait RNT is predominantly assessed using measures specific to these processes (e.g., Spinhoven et al., 2018; see Samtani & Moulds, 2017, for a recent review). Depressive rumination is characterised by a repetitive focus on “the causes, meanings, and consequences of depressive symptoms” (Nolen-Hoeksema, 1991, p. 569), and is heavily implicated in the onset and maintenance of depression (Nolen-Hoeksema, Wisco, & Lyubomirsky, 2008). Worry comprises “a chain of thoughts and images, negatively affect-laden, and relatively uncontrollable” (Borkovec, Robinson, Pruzinsky, & DePree, 1983, p. 10) and is a key characteristic of generalised anxiety disorder (GAD; Borkovec, Ray, & Stober, 1998).

Trait RNT is a relatively automatic and inflexible response style to negative states (Nolen-Hoeksema et al., 2008). It has been hypothesised that repeated and prolonged episodes of state RNT in the context of negative affect cause this to become consolidated into an inflexible pattern of responding from which it is difficult to disengage (Watkins & Nolen-Hoeksema, 2014, Watkins and Roberts, 2020). As a result, there is growing interest in preventive approaches to reducing vulnerability to episodes of state RNT, which should act to limit rehearsal of this response pattern, thereby stopping the development of maladaptive trait RNT.

Recent years have seen a rapid expansion in work to identify transdiagnostic intermediate phenotypes that constitute a risk factor for RNT (e.g., Nolen-Hoeksema & Watkins, 2011; Watkins & Roberts, 2020). The executive processes of working memory (WM) have been identified as a key candidate (Joormann, Yoon, & Zetsche, 2007; Koster, De Lissnyder, Derakshan, & De Raedt, 2011; Watkins & Roberts, 2020). These processes can be fractioned into three major functions (Miyake et al., 2000): mental set shifting (shifting), updating and monitoring of representations within working memory (updating), and inhibition of prepotent responses (inhibition^1^). Whilst there is evidence that individual differences in a unitary executive functioning construct fully account for variance in the inhibition function, it is proposed that vulnerability to psychopathology may be linked to processing-specific deficits in shifting and/or updating (Snyder, Miyake, & Hankin, 2015). As explained below, predominant theories of both worry and depressive rumination hypothesise a causal relationship between RNT and these WM processes.

Attentional Control Theory (ACT; Eysenck & Derakshan, 2011; Eysenck, Santos, Derakshan, & Calvo, 2007) specifies a series of hypotheses regarding the relationship between trait anxiety and shifting, inhibition, and updating (see Berggren & Derakshan, 2013, for a review). ACT predicts that trait anxiety is associated with impairments on measures of inhibition and shifting, and that updating may be relatively spared unless it is tested under stress or threat (Eysenck, Derakshan, Santos, & Calvo, 2007). However, this view has recently been challenged, and there is emerging evidence that trait anxiety is related to impaired updating more generally (Gustavson & Miyake, 2016). Trait anxiety is characterised by high levels of worry, and it has been suggested that attentional control difficulties play a causal role in the initiation of pathological worry (Hirsch & Mathews, 2012), and are associated with more frequent and difficult to suppress intrusions (Bomyea & Amir, 2011).

Several models of rumination propose that impairments inhibiting negative irrelevant information, shifting attention away from negative cognitions, and discarding such content from WM causally contribute to the onset and maintenance of RNT (e.g., Joormann et al., 2007; Koster et al., 2011). It is argued that these deficits may be specific to negative material (Joormann, 2010; Joormann & Gotlib, 2008; Koster et al., 2011), with a recent emphasis on impaired updating of affective information (e.g., Pe, Brose, Gotlib, & Kuppens, 2016).

Consistent with these models, substantial correlational evidence supports an association between RNT and executive functioning impairments (e.g., Berman et al., 2011; Chang, Ecker, & Page, 2017; Curci, Lanciano, Soleti, & Rime, 2013; De Lissnyder, Koster, Goubert, Onreadt, Vanderhasselt, & De Raedt, 2012; Joormann & Gotlib, 2008; Joormann, Levens, & Gotlib, 2011; Pe et al., 2016; Stefanopoulou, Hirsch, Hayes, Adlam, & Coker, 2014). However, relatively few studies have directly tested the causal nature of this association (Roberts, Watkins, & Wills, 2017). A recent systematic review concluded that the association between degraded executive functions and RNT does not represent a generalised impairment, but is instead specific to the ability to update the contents of WM by discarding information that is no longer relevant (Zetsche, Burkner, & Schulze, 2018). Consistent with this, there is evidence from twin studies of longitudinal associations between depression and executive functioning that are specific to the updating function (Friedman, du Pont, Corley, & Hewitt, 2018). A key recommendation arising from Zetsche et al.’s (2018) meta-analysis is the use of experimental manipulations to test the causal hypothesis that the ability to remove from WM information that does not serve one's current goal contributes to susceptibility to RNT.

The removal function of WM is defined as “the exclusion of information from working memory in service of the current goal” (Lewis-Peacock, Kessler, & Oberauer, 2018, p.1). There is evidence using WM updating paradigms that this is an active process that can be distinguished from decay: when participants are cued with the item to-be-updated in advance of the replacement stimulus, the participant actively removes that item in advance of the replacement operation (Ecker, Lewandowsky et al., 2014; Ecker, Oberauer et al., 2014). There are three main approaches to measuring removal of information from WM. The first of these involves a subset of information in working memory being marked as relevant (e.g., with a retro-cue) and the rest being marked as irrelevant. This should lead to faster and more accurate access to the still relevant information. An example of this is seen in the Modified Sternberg task (Joormann & Gotlib, 2008), which contrasts trials that assess interference from no-longer-relevant information with trials where such interference is not present. A second approach is to test the accessibility of the contents that was to-be-removed. A problem with this approach, however, is that participants will learn quickly that removing this information may not be necessary – and indeed may be unhelpful – if they are subsequently asked to access it. Approaches to mitigate this problem have typically only asked participants to report the removed information on a very small subset of trials. The third approach is to use an updating paradigm, where participants remove outdated associations between items and their spatial context in order to enable the creation of new ones. The removal function is a requirement of working memory updating (Ecker, Lewandowsky, Oberauer, & Chee, 2010), and a core purpose of removal is to support updating. As such, WM updating paradigms are especially well-suited to measuring and manipulating the ability to discard information from WM.

Recent years have seen a growing interest in the use of Cognitive Control Training (CCT) to examine the effects of manipulating executive functions on depressive symptomatology (Koster, Hoorelbeke, Onraedt, Owens, & ). CCT is based on the assumption that cognitive control capabilities can be improved with repeated practice (e.g., Jaeggi, Buschkuehl, Jonides, & Shah, 2011), and is typically evaluated by examining (1) near transfer: improvement on unpractised task(s) that are similar to the training task (representing the generalisation of skills across closely related domains), and (2) far transfer: improvements on outcomes that differ from the training task but are believed to rely on the construct being trained. Near transfer is thus considered to demonstrate that CCT has successfully improved the target cognitive mechanism(s), and far transfer is inferred to indicate training-related cognitive plasticity that will lead to beneficial outcomes in everyday functioning (Shipstead, Redick, & Engle, 2010). The critical component of tasks that measure or train WM is that they challenge the limits of immediate attention, thereby requiring controlled processing (Shipstead et al., 2010). Adaptive training paradigms are thus predicted to result in improvements to the underlying WM process(es) by adjusting task difficulty based on performance, such that the participant is experiencing a consistent stress on the boundaries of their WM capabilities. Importantly, only in adaptive training is the participant experiencing a consistent mismatch between task demand and supply of cognitive resource. There is evidence that this is critical to triggering cognitive plasticity and enabling change to fluid abilities (Klingberg, 2010, Lövdén et al., 2010, Pergher et al., 2019)). It has been widely recognised that a key challenge for this field is ensuring that adaptive training is contrasted with a closely matched active control group, frequently non-adaptive training using the same paradigm, which is regarded as the best type of control condition for studies of WM training (Shipstead, Redick, & Engle, 2010). This approach is advantageous because the conditions are matched on features such as materials, experimenter contact, ability to follow a schedule, use of a computer, and expectation of helpfulness (e.g., Klingburg, 2010; Pergher et al., 2019). The critical difference is that participants in the control group are not consistently training at the boundary of their capabilities. This is important both to ensuring that a similar standard for active control conditions is set across different studies, and to isolating the hypothesised mechanism of change in WM training, namely, triggering cognitive plasticity to facilitate improvements to fluid abilities.

The ability to remove information from WM is the key WM process implicated in RNT (Zetsche, Bürkner, & Schulze, 2018), and as such, a promising target mechanism for CCT to reduce RNT; susceptibility to RNT is a strong candidate for assessing clinically meaningful far transfer following CCT.

The results of studies to-date examining the effects of CCT on RNT have been mixed, and have typically focused on variants of two main training paradigms: the dual n-back task (Jaeggi, Buschkuehl, Jonides, & ), and the paced auditory serial addition task (PASAT; Gronwell, 1977). The adaptive dual n-back task presents participants with trials consisting of both visual (e.g., a square in different locations) and auditory (e.g., spoken letters) stimuli and participants are required to indicate whether each stimulus matches stimuli that appeared *n* trials previously. Task difficulty is adapted such that, based on each participant's performance, the value of *n* in the subsequent block is increased or decreased by one item (Jaeggi et al., 2008). Six studies (De Putter, Vanderhasselt, Baeken, De Raedt, & Koster, 2015; Grol et al., 2018; Hotton, Derakshan, & Fox, 2018; Onraedt & Koster, 2014; Sari, Koster, Pourtois, & Derakshan, 2016; Wanmaker, Geraerts, & Franken, 2015) did not find evidence that adaptive dual n-back training was effective in reducing rumination or anxiety, including in individuals with anxiety or depression (Wanmaker et al., 2015), and samples high in trait rumination (Onraedt & Koster, 2014). Course-Choi, Saville, and Derakshan (2017) found that seven days of dual n-back training resulted in significant reductions in worry, but not rumination, but they did not find evidence of near cognitive transfer.

Several studies have examined the effects of CCT on rumination using an adaptive version of the PASAT. The adaptive PASAT audially presents participants with a stream of digits and they are instructed to indicate the sum of the last two digits. The inter-stimulus interval between each digit is adapted based on participant performance, causing the digits to follow faster or slower. There is evidence that CCT using the PASAT reduced trait rumination in depressed patients (Siegle et al., 2007, 2014; Vanderhasselt et al., 2015), high ruminators (Hoorelbeke, Koster, Vanderhasselt, ), and patients with remitted depression (Hoorelbeke & Koster, 2017). However, a key limitation of the Hoorelbeke and Koster (2017) and Vanderhasselt et al. (2015) studies is that there was no assessment of transfer to an unpractised cognitive task, leaving the underlying cognitive mechanism for any observed differences on other outcomes ambiguous. Two studies have included a measure of transfer to an unpractised cognitive task (Hoorelbeke et al., 2015; Hoorelbeke, Koster, Demeyer, Loeys, & Vanderhasselt, 2016) and found no significant differences between their training and active control groups in improvements on working memory or dual n-back tasks. As such, these studies have yet to establish that training using the adaptive PASAT demonstrates reliable benefits to WM processes. CCT using the PASAT therefore appears to reduce rumination in at risk and clinical samples, but the cognitive mechanism underpinning these results is unclear. To our knowledge, no CCT studies to-date have used a targeted WM updating paradigm and evaluated both near transfer to an untrained paradigm, and far transfer to RNT.

In order to examine the causal impact of WM updating on RNT, it is essential that the CCT paradigm adequately isolates and targets the hypothesised mechanism of action (i.e., the ability to update WM by removing no-longer relevant information). Of the three approaches to measuring and manipulating the removal function (1. Marking a subset of information as irrelevant using a retro-cue; 2. Testing the accessibility of the contents that were to-be-removed; 3. Using a WM updating task), a WM updating task is best suited to adaptation for a WM training intervention. This is because WM training requires trial-by-trial measurement of the ability to remove irrelevant information from WM in order to vary task difficulty adaptively and continuously train participants at the limits of their capacity (Shipstead et al., 2012). The present study therefore sought to develop a novel adaptive version of an established WM updating task (Ecker et al., 2010) to test the hypothesis that improving WM updating reduces RNT. A critical strength of this paradigm is that there is evidence that it involves an active removal function (Ecker et al., 2014), and that successful execution of every trial requires the removal of no-longer-relevant information from WM.

The WM updating task comprises three major component processes: retrieval, transformation, and substitution. First, updating may or may not require the retrieval of the information to be updated if this information is not present in the updating prompt (for example, if a restaurant manager is advised “there will be five customers more than initially expected”, it is necessary to retrieve the previously expected number of customers in order to make the update). Second, updating may or may not require transformation (for example, if the restaurant manager is instead advised “actually there will now be 25 customers” no transformation is necessary to update WM). Finally, updating may or may not involve substitution (for example, if the manager is informed “three customers cancelled their reservation, but we have taken three new bookings” the total number of customers does not need to be substituted for a new value in order to update WM). Traditional WM updating tasks present a set of stimuli to be remembered and then repeatedly replace one or more items for participants to keep track of until the end of each trial, when they are asked to recall the most recent stimuli set. Ecker et al. (2010) used a series of discrete cognitive operations (distinguishing retrieval, transformation, and substitution) to specifically target each of the component processes critical to successful WM updating. The task is thus ideally suited to experimental targeting of WM updating using CCT.

Although a number of previous CCT studies have examined the effects of cognitive training on RNT, in several studies the cognitive processes targeted have either not been directly assessed using a valid measure of near transfer as a manipulation check, or there has not been evidence that the training was successful in manipulating the target cognitive processes (i.e., the absence of a near transfer effect to an unpractised task).

An important unanswered question is whether the relationship between WM updating deficits and RNT constitutes a general impairment or a more specific difficulty in manipulating negative information. It has been proposed that the association between executive functioning deficits and RNT is most clear when the stimuli used are negative, emotional, and personally relevant (Beckwe, Deroost, Koster, De Lissnyder, & De Raedt, 2014; Koster, De Lissnyder, & De Raedt, 2013). In contrast, a recent meta-analysis found that the association between RNT and deficits in executive functioning was independent of stimuli valence (Zetsche et al., 2018). However, the authors were only able to distinguish between neutral and emotional (i.e., combining both positive and negative) stimuli, and as a result, it remains unclear whether or not individuals high in RNT may experience selective impairments in processing negative content.

To examine the role of stimulus valence (negative versus neutral) in the relationship between WM updating and RNT, we compared adaptive WM updating training using negatively valenced stimuli (NEGA), adaptive WM updating training using neutral stimuli (NEUA), and a neutral non-adaptive WM updating control training (NEU) in which the training difficulty did not vary based on participant performance and so should not be as effective in training WM. Participants were randomised to 20 sessions of (i) NEUA, (ii) NEGA, or (iii) non-adaptive NEU training. Near transfer was assessed using a task that distinguishes WM updating for positive and negative stimuli. We examined far transfer using a measure of susceptibility to state RNT in response to a ruminative cue. Improvements in WM updating were hypothesised to increase efficiency in the application of cognitive control to remove unwanted ruminative thoughts, thereby supporting execution of the task at hand (in our study, a task requiring participants to focus on the breath) and reducing susceptibility to interference from state RNT.

In sum, the present study sought to examine the following hypotheses:

(1) Relative to the non-adaptive training, neutral adaptive WM updating training (hypothesis 1a), and negative adaptive WM updating training (hypothesis 1b) would improve performance on an unpractised task that measures WM updating (near transfer).
(2) Compared to the non-adaptive training, neutral adaptive WM updating training (hypothesis 2a), and negative adaptive WM updating training (hypothesis 2b) training would reduce susceptibility to state RNT (far transfer).

The focus of the study was on reducing vulnerability to episodes of state RNT in healthy young people. We therefore did not predict that our training would directly reduce pre-existing levels of trait RNT, which we expected to be below clinical levels. Secondary analyses report trait RNT levels between groups pre- and post-training to provide clarity of the pattern of data in this respect. Should our training be successful in reducing vulnerability to state RNT, then a next step would be to establish whether this could be used over a longer time period to prevent the rehearsal of RNT into a maladaptive trait response style.

## 1 Method

### 1.1 Design and power

The study had a 3 (condition) x 2 (time) double-blind randomised controlled design, with the between-subject factor of training condition, and within-subject factor of time. Ethical approval was obtained from the University of Exeter Psychology ethics committee, and written informed consent was obtained for all participants. A power calculation was conducted using G*Power to estimate the sample size required to detect a significant effect of CCT on RNT using an ANCOVA model. Based on a medium to large effect size (Hoorelbeke and Koster [2017] report f = .39 for the outcome of ruminative brooding) with power = .80, alpha level = .05, a sample size of 68 participants is required. Our novel CCT involved a substantial time commitment and its acceptability has yet to be evaluated, we therefore sought to recruit a minimum of 120 young people to allow for the possibility of relatively high levels of attrition.

### 1.2 Participants

Adolescence (defined as ages 10–24; Sawyer, Azzopardi, Wickremarathne, & Patton, 2018) is proposed to be a key developmental window in which to target proximal cognitive mechanisms implicated in the emergence of RNT. We therefore recruited young people aged 16–24 from local educational institutions (Exeter College, Exeter University) via email, and advertisements on local noticeboards, the University of Exeter website, and research participation boards. The only exclusion criterion applied was that all participants were required to be sufficiently fluent in English to comprehend the study materials. Additionally, individuals reporting suicidal risk during the baseline assessment were excluded and referred to appropriate sources of support in accordance with local clinical risk policies.

### 1.3 Working memory updating training

**Fig. 1:**
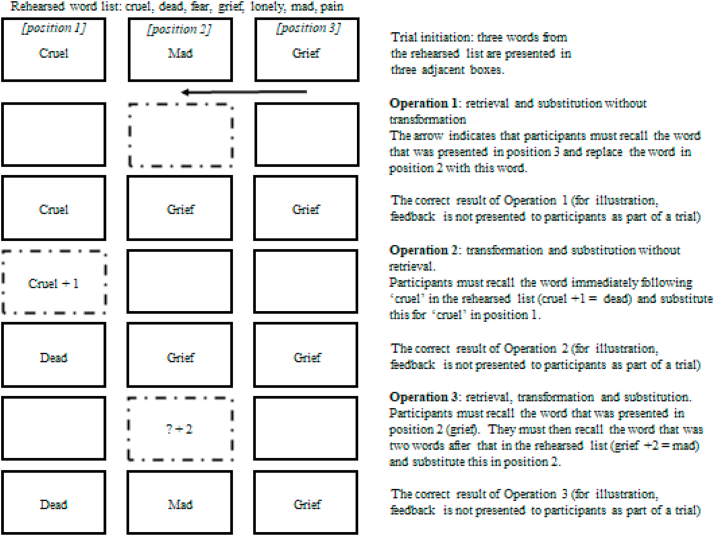
An example trial using negative stimuli.

The WM training was developed from Ecker et al.’s (2010) task. Each training session comprised an initial word-list rehearsal phase, followed by the training task. The training task involved completing a series of trials where participants practised cognitive operations that required the three working memory updating processes (see Fig. 1 for examples). All trials require the removal of outdated associations between words and their spatial context. At the end of each trial, a test question assessed participant accuracy in successfully executing the cognitive operations for that trial. The training task began with two practice trials involving two operations each. In the adaptive conditions, the number of cognitive operations within a trial then varied based on participant performance (with greater accuracy on the test questions resulting in subsequent increases in the number of operations per trial). In the non-adaptive condition, each trial then involved completing three cognitive operations, and this did not vary according to how accurate participants were in responding to the test questions. Participants in the adaptive conditions completed five blocks of five trials per training session, and participants in the non-adaptive condition completed 10 blocks of five trials per training session. Participants were asked to complete a total of 20 training sessions over 28 days. There was an interval of 2.5 s between trials, and no breaks were provided between blocks.

#### 1.3.1 Word rehearsal phase

At the beginning of each training session participants were presented with a list of seven words, which they spent 1 min rehearsing. Words were randomly selected from a pool of 35 negative or 35 neutral words taken from the Affective Norms for English Words battery (ANEW; Bradley & Lang, 1999) and matched on length and frequency across conditions. Participants completed 2 min of cued recall where two letters from each word are presented and they typed in the letters to complete the words in the order in which they were presented during rehearsal. Finally, they were tested on free recall to the criterion of recalling the words in the order in which they had been presented at rehearsal with 100% accuracy. A new set of seven words of the same valence was presented on each training session. Words selected for each new session could not have been used in the training session immediately before that day's training. Each training session concluded with a final test of recall of word order for the day's stimulus set as memory check.

#### 1.3.2 Cognitive operations targeted within the training

The training required participants to practise removing outdated associations between words and their spatial context. This was done by completing a series of cognitive operations that involved the three WM updating processes (substitution, transformation, or retrieval). Participants were presented with an initial set of three words from the list they had rehearsed (Fig. 1). A red frame was then presented around one of these words to indicate that was the word for which they would perform a cognitive operation. All operations required substitution (S), meaning that the word in the frame was substituted with a new word. Transformation (T) involved counting forwards through the rehearsed word list by either +1 or +2, starting from the word in the red frame (e.g., in Fig. 1, operation 2: participants start with the word “cruel” in the red frame and count forwards one through the rehearsed list, to get to the word “dead”). Retrieval (R) required participants to retrieve the word in the red frame from memory (i.e., the red frame did not contain the word to be used, see Fig. 1, operations 1 and 3). In operations without retrieval, the word was provided (e.g., Fig. 1, operation 2). Trials comprised a series of consecutive operations with no feedback between operations.

#### 1.3.3 Training task structure

Each trial was initiated by a key press. The starting words were presented in their frames, all at once, for 1 s. Then the instruction for the first cognitive operation was displayed and participants were required to type the result within 5 s. If no response was made within the time limit, an error was recorded and a new instruction was presented for the next operation. After all the operations for that trial were completed, a test question was presented: a single test word was presented in one of the frames, and participants were asked indicate whether this was the correct word for that frame (Y/N) within a 5 s time limit. For example, in Fig. 1, the correct word in the left frame at the end of the operations is “dead”. On 40% of trials, the correct answer to the test question was yes (Y; i.e., the word presented was the correct word for that frame). On 60% of trials, the correct answer was no (N). Where trials involved completing more than two cognitive operations, these test questions presented the word that had appeared in the test frame on the third cognitive operation before the question (e.g., the word that had appeared in that frame at operation 1 in Fig. 1: “cruel” was in the left frame at operation 1, and so if the test question presented “cruel” in the left frame instead of “dead” then the response would be no (N), that was no longer the correct word for that frame). As a result, in order to answer the question correctly, participants could not rely on familiarity of the word having appeared in that location, and must successfully execute the cognitive updating operations for that trial. Where trials only involved two cognitive operations, the test question presented a word from a consecutive frame in the test frame (e.g., in Fig. 1, presenting “mad” in the left frame is not correct because this was the word from the middle frame).

#### 1.3.4 Performance-based variation in training difficulty

In the adaptive training condition, for each block of five trials, if responses to the test questions for three or more of the trials were correct, then in the next block each trial would involve completing one more cognitive operation. If this criterion was not met, then each trial in the next block involved completing one fewer cognitive operation. If the trials already involved the minimum number of cognitive operations, which was two, then failure to answer three test questions correctly resulted in the next block continuing to present two cognitive operations per trial. Each new training session began at one below the maximum number of cognitive operations for which three or more trials within a block were correct on the previous day.

### 1.4 Outcome measures

#### 1.4.1 Cognitive (near) transfer: Modified Sternberg task

**Fig. 2:**
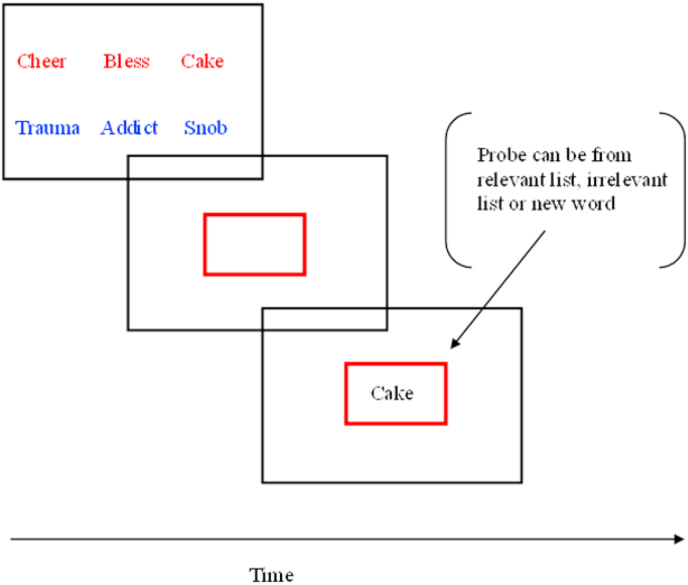
An example trial in the modified Sternberg task.

A shortened version of the modified Sternberg task (Joormann & Gotlib, 2008) was used to index WM updating for positive and negative words. Participants were presented with two lists of three words simultaneously, which they were instructed to remember (Fig. 2). The word lists could be of positive or negative valence. A cue was then presented indicating which list would be relevant for evaluating the probe word. Finally, a single word (the probe) was presented and participants were asked to indicate whether this word belonged to the relevant (cued) list. The probe word could be from the relevant list, the irrelevant list, or be a new positive or negative word.

Trials were organised into three continuous blocks, with each trial type occurring four times per block, resulting in 12 trials per condition over a duration of approximately 30 min. Depressed participants have been found to take significantly longer to correctly reject negatively-valenced irrelevant words, which is interpreted as indexing impairments in discarding negative material from WM (Joormann & Gotlib, 2008). Rumination-related errors on the modified Sternberg task are typically low (Joormann & Gotlib, 2008; Roberts, 2013), with experimentally-induced changes to performance observed in alterations to efficiency of responding. The primary dependent variables were thus reaction times to reject new negative words and negative words from the irrelevant list. Errors were additionally analysed to confirm the absence of differential speed-accuracy trade-offs between groups.

#### 1.4.2 Far transfer: state RNT

Susceptibility to state RNT in response to a stressor was assessed using the breathing focus task (Hirsch, Hayes, & Mathews, 2009). This was administered immediately following a goal-cueing procedure that has been shown to elicit state RNT (Roberts, Watkins, & Wills, 2013). The goal-cueing procedure instructed participants to identify an ongoing and unresolved concern that had repeatedly come into their mind and caused them to feel negative or stressed during the previous week. Participants worked through a 10 min pre-recorded script delivered over headphones, which prompted them to focus on the concern identified (see Roberts et al., 2013). Items in the script included “think about what is important about this difficulty in terms of your personal goals” and “focus on how this problem reflects a lack of progress on important personal goals”. This procedure has been demonstrated to reliably elicit state RNT (Kirschner et al., 2019; Roberts et al., 2013; Roberts et al., 2020), with individuals higher in trait rumination reporting greater RNT following the cue.

The breathing focus task (BT; Hirsch et al., 2009) was administered immediately after the goal-cueing procedure. During the 5 min breathing focus period, participants were instructed to focus their attention on their breathing, and a computer-generated tone was presented 12 times at random intervals of between 20 and 30 s. At each tone participants were required to indicate whether they had been focusing on their breathing immediately before the tone or if they had experienced an intrusion. Response options were (a) breathing, (b) physical sensations, (c) the problem focused on in the previous task, or (d) other thoughts. If a thought intrusion was reported, then the participant was required to rate this as negative, positive, or neutral in emotional tone. The total number of negative intrusions constituted the participant's RNT score (Hirsch et al., 2009).

#### 1.4.3 Other measures

The Ruminative Responses Scale of the Response Styles Questionnaire (RRS; Nolen-Hoeksema & Morrow, 1991) is a 22-item measure of trait depressive rumination. Items include “analyze recent events and try to understand why you are depressed” and “think about how alone you feel”. Responses range from 1 (*almost never*) to 4 (*almost always*) for what participants “generally do” when they are feeling sad or depressed. Item scores are summed to generate an overall score (range: 22–88); higher scores represent a greater trait tendency to depressive rumination. The RRS has high internal consistency, acceptable construct validity, and good test-retest reliability (Nolen-Hoeksema & Morrow, 1991; Treynor, Gonzalez, & Nolen-Hoeksema, 2003).

The Penn State Worry Questionnaire (PSWQ; Meyer, Miller, Metzger, & Borkovec, 1990) is a 16-item measure of trait worry. Respondents rate the extent to which each item applies to them on a 5-point scale ranging from ‘not at all typical of me’ (1) to ‘very typical of me’ (5). Scores range from 16 to 80, with higher scores reflecting greater trait worry. The PSWQ has demonstrated good internal consistency, validity, and reliability (Brown, 2003; Hazlett-Stevens, Ullman, & Craske, 2004).

The Patient Health Questionnaire (PHQ–9, Kroenke, Spitzer, & Williams, 2001) is a nine–item measure designed to assess the severity of depression symptoms over the past two weeks. Scores range from 0 to 27, with greater scores indicating more severe symptoms of depression. It has good internal consistency and construct validity (Kroenke et al., 2001).

The Backward Digit Span (BDS) is one of the core WM subtests of the Wechsler Adult Intelligence Scale (WAIS-IV UK), and is interpreted as an index of WM capacity. Participants hear a sequence of digits read aloud at a rate of one digit per second and are required to recall the sequence correctly in reverse order. Each trial consists of two sequences and sequence length increases progressively with each trial. Administration is discontinued when both items from a given pair are failed. The WAIS-IV UK subtests are widely used and possess good psychometric properties (Cullum & Larrabee, 2010, pp. 167–187).

The acceptability of the training was measured using a series of brief Likert-type scales, in which participants indicated on a scale from 1 (*strongly disagree*) to 5 (*strongly agree*) the extent to which they (a) found the training easy to understand, (b) found the training effortful to complete, (c) felt happy about completing the training, (d) found it easier to concentrate following the training, and (e) felt less bothered by negative thoughts following the training. A free text box provided the opportunity to give additional feedback.

### 1.5 Procedure

Participants were invited to complete a baseline assessment at the University of Exeter. They were informed that the research was examining whether repeated practice at a computerised cognitive training improves performance on unpractised cognitive tasks, and provided written informed consent. Participants then completed the baseline cognitive and self-report measures, before receiving the training instructions and registering to the online training platform. Randomization was built into the registration by an independent computer programmer using an automated randomization code. Participants completed 20 online CCT sessions, during which they were able to contact the researcher who completed their baseline assessment with questions or technical concerns. On average, participants completed 14 days of training (*SD* = 8, range: 0–23), see Table 1. The post-training assessment was completed by a second researcher who remained blind to the training condition. Participants repeated the self-report and cognitive assessments and provided feedback on their experience of the cognitive training using a brief acceptability questionnaire. All participants were debriefed and compensated for their time.

**Table 1:** Group characteristics by training condition.

Variables	NEU			NEUA			NEGA			Over all		
	*n* (follow up *n*)	Baseline Mean (*SD*)	Follow-up Mean (*SD*)	*n* (follow up *n*)	Baseline Mean (*SD*)	Follow-up Mean (*SD*)	*n* (follow up *n*)	Baseline Mean (*SD*)	Follow-up Mean (*SD*)	*N* (follow up *N*)	Baseline Mean (*SD*)	Follow-up Mean (*SD*)
Age	42	19.02 (1.76)	–	38	19.42 (1.83)	–	44	19.66 (2.26)	–	124	19.37 (1.97)	–
PHQ-9	42 (29)	6.1 (4.19)	6.1 (3.72)	38 (26)	5.68 (3.72)	6.42 (4.88)	43 (30)	5.67 (3.6)	6.17 (4.42)	123 (85)	5.82 (3.82)	6.22 (4.3)
RRS rumination	42 (28)	44.62 (13.13)	44.46 (12.35)	38 (25)	44.24 (12.24)	44.48 (13.73)	44 (30)	43.25 (9.71)	44.43 (11.41)	124 (83)	44.02 (11.66)	44.46 (12.31)
PSWQ	42 (29)	47.26 (15.63)	47.93 (15.07)	38 (26)	49.71 (14.11)	50.46 (14.57)	44 (29)	51.45 (11.77)	51.03 (11.7)	124 (84)	49.5 (13.88)	49.79 (13.74)
RRS Brooding	42 (28)	9.5 (3.68)	9.32 (3.49)	38 (26)	10.47 (3.38)	9.81 (3.38)	44 (30)	10.25 (2.9)	10.73 (3.51)	124 (84)	10.06 (3.33)	9.98 (3.47)
BT score	42 (28)	1.76 (1.85)	2.32 (1.91)	37 (25)	2.35 (2.06)	1.80 (2.04)	43 (27)	2.63 (2.30)	1.81 (1.62)	122 (80)	2.25 (2.09)	1.99 (1.85)
Backward Digit Span	42 (29)	9.6 (2.21)	11.03 (2.16)	38 (26)	10.03 (2.21)	11.15 (3.07)	44 (30)	10.75 (3.01)	12.07 (3.58)	124 (85)	10.14 (2.55)	11.44 (3)
RT negative irrelevant	42 (28)	1560.02 (643.60)	1149.64 (457.98)	38 (26)	1390.68 (577.50)	926.22 (297.84)	44 (30)	1528.26 (575.53)	1075.72 (400.27)	124 (84)	1496.86 (599.49)	1054.09 (399.12)
RT new negative	42 (28)	1038.45 (357.90)	908.52 (356.37)	38 (26)	965.91 (306.22)	741.47 (205.26)	44 (30)	1058.38 (368.47)	809.25 (262.49)	124 (84)	1023.29 (346.27)	821.36 (287.56)
Errors negative irrelevant	42 (28)	1.05 (1.13)	0.96 (1.00)	38 (26)	1.00 (0.96)	0.85 (1.05)	44 (30)	1.07 (1.17)	0.93 (1.05)	124 (84)	1.04 (1.08)	0.92 (1.02)
Errors new negative	42 (28)	0.07 (0.26)	0.11 (0.31)	38 (26)	0.13 (0.41)	0.08 (0.27)	44 (30)	0.25 (0.87)	0.10 (0.31)	124 (84)	0.15 (0.58)	0.10 (0.30)
RT positive irrelevant	42 (28)	1581.90 (618.14)	1160.71 (430.52)	38 (26)	1352.60 (535.20)	992.30 (393.58)	44 (30)	1498.51 (554.93)	1041.10 (321.87)	124 (84)	1482.04 (574.34)	1065.87 (384.84)
RT new positive	42 (28)	1050.79 (430.60)	842.10 (234.71)	38 (26)	985.55 (291.54)	749.42 (184.71)	44 (30)	1056.44 (358.59)	804.34 (311.48)	124 (84)	1032.80 (365.14)	799.93 (252.00)
Errors positive irrelevant	42 (28)	1.00 (1.13)	0.46 (0.88)	38 (26)	1.18 (1.31)	0.73 (0.83)	44 (30)	1.11 (1.06)	0.73 (0.83)	124 (84)	1.10 (1.16)	0.64 (0.85)
Errors new positive	42 (28)	0.12 (0.63)	<.001 (<0.001)	38 (26)	0.08 (0.27)	0.15 (0.37)	44 (30)	0.23 (0.80)	0.07 (0.25)	124 (84)	0.15 (0.62)	0.07 (0.26)
Completed training days	42	13.48 (7.95)	–	38	14.03 (7.33)	–	44	13.36 (7.62)	–	124	13.6 (7.59)	–
Mean training time (minutes and seconds)	42	22.38 (8.61)	–	38	25.89 (10)	–	44	24.25 (13.58)	–	124	24.12 (11.01)	–

### 1.6 Data analytic plan

Analyses were conducted using IBM SPSS Statistics 25. Baseline characteristics by training groups are presented using means and standard deviations (Table 1). Data are analysed on an intention-to-treat basis (as randomised) based on case outcome data, and missing data are assumed to be missing at least at random (MAR).^2^ No imputation of data was performed.

#### 1.6.1 Cognitive (near) transfer

To examine near cognitive transfer, group comparisons were conducted on RTs to new and intrusion words (words from the irrelevant list) on the modified Sternberg task using analysis of covariance (ANCOVA) models adjusted for their baseline scores (e.g., RT to correctly reject negative intrusion words at follow-up adjusted for RT to correctly reject negative intrusion words at baseline), with NEU as the reference group. We thus constructed ANCOVA models, with training condition (Hypothesis 1a: NEUA vs. NEU; Hypothesis 1b NEGA vs. NEU) as the independent variable, examining cognitive transfer on the separate outcomes of: (1) RT to correctly reject negative intrusion words, and (2) RT to correctly reject new negative words. There were thus two dependent variables for each primary hypothesis. Error rates were additionally examined to confirm that our intervention had not differentially influenced speed-accuracy trade-offs between groups. We also report RTs and errors to positive words to confirm that any effects of training were specific to negative stimuli, in line with our predictions. Planned comparisons examined differences between each of the adaptive training conditions and the reference group. A 90% winsorisation was applied to individual participants’ reaction time data points to reduce the influence of extreme values. This was applied at the intra-individual level, such that the 5% most extreme reaction times in each tail were corrected to the 5th and 95th percentile respectively.

#### 1.6.2 Far transfer (state RNT)

Planned group comparisons were made for the outcome of state RNT using an ANCOVA model adjusted for baseline scores, comparing first NEUA and then NEGA with NEU as the reference group. We additionally adjusted the model for individual differences in participant ratings of the severity of the ruminative problem cued in the RNT stressor task.

#### 1.6.3 Secondary analyses

Planned group comparisons were made for the outcomes of trait rumination (RRS) and trait worry (PSWQ) using an ANCOVA model adjusted for baseline scores, comparing first NEUA and then NEGA with NEU as the reference group.

PROCESS (Hayes, 2013) was used to construct separate moderation models to examine whether trait RNT (RRS and PSWQ) moderated the effect of each of the adaptive trainings (NEUA and NEGA), compared to NEU, on WM updating outcome scores on the modified Sternberg task after adjusting for baseline WM updating scores. Bootstrapping was used to generate standard errors and confidence intervals by bootstrapping the samples n = 5000 times.

## 2 Results

### 2.1 Sample characteristics

**Fig. 3:**
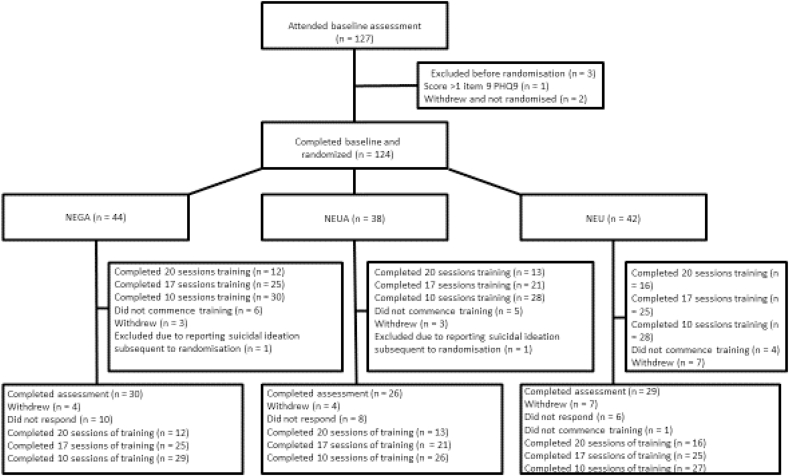
Consort diagram for the flow of participants.

124 participants were randomly allocated to non-adaptive (NEU: *n* = 42), neutral adaptive (NEUA, *n* = 38), or negative adaptive (NEGA, *n* = 44) CCT (see Fig. 3 for the CONSORT participant flow diagram). 85 participants completed the post-training assessment (NEU: *n* = 29, NEUA: *n* = 26, NEGA: *n* = 30). The mean number of training days completed was 13.6 (NEU: *M* = 13.48, NEUA: *M* = 14.03, NEGA: *M* = 13.36; Table 1).

Table 1 presents participant characteristics by training condition. Acceptability ratings for the training were positive: averaged across conditions, participants endorsed that they were happy about completing the training (*M* = 4.01 out of a maximum rating of 5, *SD* = 0.67), the training was generally perceived to be easy to understand (*M* = 4.31, *SD* = 0.68), and somewhat effortful to complete (*M* = 3.57, *SD* = 0.97), participants tended to endorse finding it easier to concentrate (*M* = 3.45, *SD* = 0.86), and being less bothered by RNT (*M* = 3.20, *SD* = 0.76) following training.

### 2.2 Cognitive (near) transfer

After adjusting for baseline scores, there was a significant effect of training condition on RTs to correctly reject new negative words, *F* (2, 80) = 3.57, *p* = .033, partial η^2^ = 0.08. Relative to the NEU condition, people in the NEUA condition had significantly faster RTs to correctly reject new negative words following the training, Δ = −147.92 ms, *p* = .01, 95% CI [-258.75, −37.09], r = 0.28. There was a tendency in the same direction for RTs to correctly reject negative intrusions, with participants in the NEUA condition being faster than those in the NEU condition,^3^ Δ = - 171.50 ms, *p* = .054, 95% CI [-346.10, 3.10], r = 0.21. After adjusting for baseline scores, the NEUA condition did not significantly differ from the NEU condition in error rates, new negative: Δ = - 0.10, *p* = .71, 95% CI [-0.65, 0.44], r = 0.04, negative intrusions: Δ = - 0.03, *p* = .71, 95% CI [-0.16, 0.16], r = 0.04. There was no evidence that the NEGA condition significantly differed from the NEU condition in either the speed or accuracy of responses to either new or intrusion negative words (all *p*s > .11). There were no significant effects of training condition on either RTs or errors to positive words (all *p*s > .08), and no significant differences between either of the adaptive training groups and the NEU group in RTs to new or intrusion positive words (all *p*s > .13). Unexpectedly, after adjusting for baseline scores, people in the NEUA condition made significantly more errors in responding to new positive words relative to people in the NEU condition, Δ = 0.15, *p* = .03, 95% CI [0.16, 0.29], r = 0.24. There were no other significant group differences for errors to new or intrusion positive words (all *p*s > .23). Thus, there was evidence that, following training, people in the NEUA condition showed improved efficiency in the processing of negative irrelevant stimuli, as compared to the NEU condition. In contrast, there was no evidence that NEGA training improved the efficiency of WM updating, as assessed using the modified Sternberg task.

### 2.3 State RNT (far transfer)

After adjusting for baseline scores, there was a significant effect of training condition on state RNT, *F* (2, 74) = 3.19, *p* = .047, partial η^2^ = 0.08. The NEUA group had significantly lower levels of state RNT post-training as compared to the NEU group, Δ = −1.11, *p* = .01, 95% CI [-2.00, −0.22], r = 0.29. The corresponding contrast was not significant for the NEGA group, Δ = −0.67, *p* = .12, 95% CI [-1.53, 0.18], r = 0.18. Thus, there was evidence that people in the NEUA condition, but not those in the NEGA condition, showed reduced susceptibility to state RNT after the training, compared to the NEU group.

### 2.4 Secondary analyses: the effects of training on trait RNT

As expected, after adjusting for baseline scores, neither the NEUA (Δ = −0.39, *p* = .87, 95% CI [-5.19, 4.40], r = 0.02) nor the NEGA (Δ = 1.01, *p* = .66, 95% CI [-3.57, 5.60], r = 0.05) group significantly differed from the NEU group on post-training trait rumination, as measured using the RRS. Likewise, neither training group significantly differed from the NEU group on post-training trait worry, as measured using the PSWQ (NEUA: Δ = −1.79, *p* = .31, 95% CI [-5.29, 1.72], r = 0.11; NEGA: Δ = −1.33, *p* = .44, 95% CI [-4.74, 2.08], r = 0.09).

### 2.5 Secondary analyses: the interaction of trait RNT and training condition on improvements to WM updating

There was no evidence that trait RNT interacted with WM updating training condition to predict improvements on the modified Sternberg task. After adjusting for baseline scores, the interaction of training condition and RRS on negative intrusions (NEUA: *F* (1, 49) = 0.30, *p* = .59, 95% CI [-11.55, 20.15]; NEGA: *F* (1, 53) = 1.28, *p* = .26, 95% CI [-28.84, 8.03]) and new negative words (NEUA: *F* (1, 49) = 1.36, *p* = .25, 95% CI [-4.03, 15.14]; NEGA: *F* (1, 53) = 0.05, *p* = .82, 95% CI [-12.98, 10.34]) was not significant. The interaction of training condition and PSWQ on negative intrusions (NEUA: *F* (1, 49) < 0.01, *p* = .97, 95% CI [-12.95, 13.52]; NEGA: *F* (1, 53) = 1.34, *p* = .25, 95% CI [-23.81, 6.38]) and new negative words (NEUA: *F* (1, 49) = 1.42, *p* = .24, 95% CI [-3.33, 13.06]; NEGA: *F* (1, 53) = 0.63, *p* = .43, 95% CI [-5.95, 13.70]) was also not significant. The same pattern was also observed for positive words (all *p*s > .09).

## 3 Discussion

The present study sought to test the hypotheses that training WM updating would (1) result in improvements in WM updating as assessed using an unpractised task (near transfer), and (2) reduce vulnerability to state RNT in response to a stressor (far transfer). We examined these effects when the training involved processing negative versus neutral stimuli. This proof-of-concept study was conducted in a sample of healthy young people, and focused on reducing episodes of state RNT. We therefore did not predict that our training would reduce trait levels of pathological RNT, which we expected to be relatively low.

Adaptive WM updating training using neutral stimuli significantly reduced times to correctly reject negative irrelevant stimuli on an unpractised WM updating task, as compared to training using a non-adaptive control task (evidence of near transfer). Moreover, these effects generalised such that participants showed reduced susceptibility to state RNT post-training (far transfer). The findings are thus consistent with the hypothesis that adaptive WM updating training using neutral stimuli improves the ability to remove negative irrelevant material from WM thereby causally reducing susceptibility to state RNT. Contrary to predictions, there was no evidence of either near or far cognitive transfer for adaptive WM updating training using negative stimuli; the hypothesis that our NEGA training would reduce susceptibility to RNT was thus unsupported. We found no evidence that trait RNT moderated the effects of our training on improvements to WM updating.

This proof-of-concept study provides preliminary evidence that training to improve WM updating may hold potential to reduce vulnerability to stress-induced state RNT. In a healthy sample of young people, we found evidence that our WM updating training resulted in significant improvements to WM updating, and reductions in vulnerability to state RNT following a stressor, which may be an important precursor to the emergence of maladaptive trait RNT and increased risk of psychopathology. The results are thus consistent with Zetsche et al.’s (2018) hypothesis that WM updating is important to understanding the causal nature of the associations between executive functioning impairments and RNT. Relatively little research in this area has focused on the susceptibility to engage in state RNT, and recent developmental models indicate that this may be especially important in understanding the emergence of ruminative habits in young people (Shaw, Hilt, & Starr, 2019; Watkins & Roberts, 2020).

Models of the emergence of ruminative habits (Shaw et al., 2019; Watkins & Roberts, 2020) suggest that rumination initially occurs as a goal-directed response to stressors, but over time repetition can lead to the automatic association of ruminative cues, such as negative mood, with engaging in maladaptive rumination as a habitual response style. Cognitive control is hypothesised to play a critical role in determining the extent to which vulnerable individuals perseverate in rumination following a stressor, thereby facilitating the rehearsal and reinforcement of the stimulus-response associations between negative mood and rumination. This would suggest that CCT to reduce vulnerability to episodes of state RNT may be of greatest benefit to young people who have not yet developed pathological ruminative habits, because the targeted deployment of WM updating processes to shift away from negative self-referent thinking in the context of a stressor may help to prevent such habits from emerging (see Shaw et al., 2019; Watkins & Roberts, 2020).

Although our training was effective in reducing vulnerability to stress-induced state RNT, we did not expect that over the time-scale of our study this would generalise to the more pathological processes observed in clinical populations. Relatively few participants in our sample reported clinical levels of pathological trait RNT, and as we predicted, our WM updating training did not significantly alter trait RNT. It may be that this standalone online CCT package is more appropriate to support healthy young people managing periods of increased stress and/or vulnerability to state RNT in order to reduce risk of psychopathology. An important next step will be to establish whether this could be used over a longer time period to prevent the rehearsal of RNT into a maladaptive trait response style. Further research in clinical groups is needed to clarify whether our findings might extend to individuals experiencing psychopathological symptoms.

We did not find evidence that baseline levels of trait RNT moderated the effect of our training on improvements to WM updating. This may partially reflect the relatively low levels of trait RNT in our sample, which resulted in a restricted range on these measures (e.g., 70% of our sample reported low or normal levels of trait rumination, with only 11% reporting rumination within the clinical range). Future studies examining the impact of our training across a range of levels of baseline trait RNT and cognitive function may be particularly helpful in elucidating whether it has greatest potential at particular thresholds on these variables.

From a basic processes perspective, the current findings make an important contribution to elucidating the causal nature of the associations between executive functioning deficits and RNT. There has been considerable debate as to whether such impairments represent a general executive functioning deficit or are specific to particular processes (e.g., Snyder et al., 2015). Following Zetsche et al.’s (2018) review, our results suggest that the specific role of updating in RNT may be an important avenue for further research. Whilst there has been considerable theoretical elaboration and correlational research on this topic, it is only recently that CCT studies have begun to directly test the hypothesis that executive functioning impairments have a causal effect on RNT. Consistent with a number of key theoretical accounts, we found evidence that manipulating WM updating reduced RNT: relative to a non-adaptive control training, WM updating training resulted in improved WM updating for negative material and lowered state RNT to a stressor. A strength of this design is that we directly measured changes in susceptibility to state rumination to a stressor, thereby overcoming a limitation of previous designs that have relied on self-report measures of trait level tendencies averaged over time. In order to establish mechanistic evidence regarding causal factors implicated in the onset and development of pathological RNT habits, it is important to elucidate whether putative mechanisms can be demonstrated to result in increases/decreases to the target process prior to pathological habits having become established. In this instance, this requires studies examining whether improving working memory updating reduces momentary susceptibility to RNT to a stressor, and whether these effects are observable in individuals who have not yet developed pathological levels of trait RNT. This approach additionally permits greater disentanglement of the inter-relations between WM updating and state-level variance in RNT to a stressor, from the role of psychopathological symptoms and processes that are highly correlated with trait RNT (e.g., depressed and anxious mood, impaired sleep and concentration, etc.). As such, the use of experimental tasks and ecological momentary assessment approaches to measure changes in RNT following CCT are important to establishing proof-of-concept of WM updating as a putative mechanism underpinning the emergence of problematic RNT.

A key limitation of the current findings is that we did not include multiple post-training assessment points, which would permit more sophisticated mechanistic analyses using improvements on the primary cognitive outcome measures (i.e., the modified Sternberg task) post-training to predict subsequent reductions in susceptibility to state RNT. As a proof-of-concept study, these promising early findings provide the basis for further research including multiple follow-up periods to examine the mediating effects of near-transfer immediately post-training on subsequent measures of far-transfer. It is of note that whilst our NEUA condition demonstrated a significant reduction in state RNT relative to the NEU condition, the NEUA condition showed an absolute reduction in RNT of approximately 5% during a 5-min sampling period, whilst the NEU condition reported a small unexpected absolute increase in RNT (Table 1). There was no evidence of statistically reliable baseline differences, or of an effect of the NEU condition on our hypothesised mechanism of change (WM updating), the groups did not differ in key prognostic variables or levels of attrition, and there is no theoretical basis to predict that the NEU condition would impair self-regulation or increase susceptibility to RNT. As such, we believe that the most reliable inference from this pattern of results supports our a priori hypotheses via the observed improvement on our mechanism of change. However, future research will be important to replicate this.

Our findings regarding the role of training stimulus valence are surprising, given the substantial evidence that RNT is correlated with both deficits and biases in the efficient processing of negative stimuli (see Koster et al., 2011). It is interesting to note that whilst the effects of WM updating training on RNT were only observed for the adaptive training using neutral stimuli, there was evidence that the training showed cognitive transfer to WM updating abilities for negatively valenced stimuli. Since our study used a healthy sample, one possible interpretation of this could be that participants experienced training using negative stimuli as less consistent with their cognitive set. Future studies comparing training in clinical and healthy samples, and involving more sophisticated measures of engagement, such as eye tracking and pupillary response, may be important to elucidating such possibilities.

Overall, participants rated the training positively, reporting that they were in general happy to complete the home practice, although found it somewhat effortful. However, these measures were administered post-training, and 39 participants declined to attend a follow-up assessment, suggesting that we may not have captured some important barriers to completing the study within our design. It is possible that the substantial time commitment involved was experienced as challenging for some of our sample of healthy young people, who were nearly all engaged in full-time education. Important unanswered questions remain regarding the optimal dosing and scheduling of CCT to reduce or prevent depression (Koster et al., 2017), and there is likely to be a trade-off between maximising cognitive benefits (through potentially longer/more challenging training regimes) and maximising acceptability and uptake. Future research will be important to address this question, and ensure that any barriers to completing CCT are adequately captured amongst those participants who discontinue from training early. Designs involving a longer follow-up period and in-depth qualitative interviews to elucidate barriers and facilitators to completing the training and follow-up assessments will be especially valuable in this respect.

Although we believe our study has a number of strengths, it is important to note several limitations. First, the absence of longer follow-up periods means that it is not possible to establish to what extent the benefits of our WM updating training were maintained over an intermediate or longer trajectory. This additionally leaves ambiguous the potential that initial effects in reducing dispositional RNT may subsequently translate to reduced vulnerability to symptoms of depression or anxiety. As a proof-of-concept study, our primary goal was to establish the potential of WM updating training to reduce susceptibility to state RNT, and a longer-term evaluation will be an important next step. Second, our WM updating training involved multiple components (every trial required an active removal function, and retrieval, transformation, and substitution were each invoked on a subset of trials). This design was intended to equally target the different updating components whilst requiring participants to remove outdated WM representations on every trial. Thus, although our focus was on targeting the ability to update WM by removing no-longer relevant information, we did not quantify the relative contribution of the task components to reducing RNT. A more fine-grained analyses of the relationship between RNT and each component on WM updating will be a helpful avenue for future investigation. Third, our measure of cognitive transfer assessed WM updating for emotional but not neutral stimuli, and we found evidence that the training using neutral stimuli improved updating for negative but not positive stimuli. As such, this leaves the role of stimulus valence somewhat ambiguous, and the possible role of neutral stimuli at the level of cognitive transfer has yet to be tested. Future research including a neutral condition within the transfer task, as well as a measure of engagement with the training stimuli is necessary in order to disentangle the possible roles of stimulus engagement and stimulus valence in these findings. Fourth, the use of a healthy adolescent sample leaves open the question of whether these effects would generalise to clinical populations, and this will be an important avenue for investigation. Our study focused on the potential of WM updating training as a preventive approach to reducing susceptibility to RNT, and a relatively small proportion of our sample reported clinically significant levels of trait maladaptive RNT (for example, 32 participants scored >50 on the RRS, with only 13 scoring >60). It will therefore be particularly important for further research to determine if our intervention may have added (or reduced) benefits for young people already experiencing pathological levels of trait RNT. Finally, the focus of our study was on reducing vulnerability to episodes of state RNT, and we therefore selected the breathing task as our measure of far transfer. Whilst this approach has a number of strengths, the psychometric properties of the breathing task are yet to be established, and past research has primarily used it to assess changes to state RNT within-session. Further research using multiple approaches to assessing state RNT will therefore be valuable.

Our study constitutes a promising first step in elucidating the causal role of WM updating in RNT. Future studies in this area could extend their scope of measurement beyond pen-and-paper assessments in the laboratory to consider the psychophysiological concomitants of the observed reductions to state RNT, in addition to examining to what extent these findings may translate beyond the laboratory using ecological momentary assessment designs (e.g., Hoorelbeke et al., 2016). Moreover, whilst the present study focused on evaluating reductions in negative clinical indicators, future research may wish to consider positive outcomes that might be associated with CCT, such as goal pursuit and goal progress, adaptive self-regulation, and positive reappraisal following a stressor.

In sum, we demonstrated proof-of-concept that WM updating plays a causal role in susceptibility to state RNT following a stressor, and found preliminary evidence to suggest that CCT may be a promising approach to reducing vulnerability to RNT in young people. The findings await replication, and a longer-term follow-up is required in order to establish how robust these effects are over time.

## Funding

This work was generously supported by a Wellcome Trust Institutional Strategic Support Award (WT097835MF).

## CRediT authorship contribution statement

**Henrietta Roberts:** Conceptualization, Methodology, Formal analysis, Writing – original draft, Funding acquisition. **Mohammod Mostazir:** Formal analysis, Writing – review & editing. **Nicholas J. Moberly:** Writing – review & editing. **Edward R. Watkins:** Writing – review & editing. **Anna-Lynne Adlam:** Conceptualization, Methodology, Writing – review & editing, Funding acquisition, Supervision.

## Declarations of competing interest

None.

## Footnotes

1 The definition and scope of the inhibition construct is the subject of debate (e.g., MacLeod, Dodd, Sheard, Wilson, & Bibi, 2003; Roberts et al., 2017). We adopt Miyake et al.’s (2000) more narrow definition, which reserves the term inhibition to describe deliberately stopping a prepotent response, for example, on the stop-signal task. We therefore do not use inhibition to describe phenomena such as interference arising as a result of incomplete deactivation of a no-longer relevant memory trace, although we note there are alternative perspectives (Joormann, 2010).

2 Missing data analyses supported this assumption. There was no evidence of systematic patterns of missingness based on key prognostic variables (e.g., baseline RNT, WM updating) or participant characteristics (e.g., gender, education). Little's MCAR test (Little, 1988) testing the null hypothesis that data are missing completely at random was non-significant (χ^2^ (64) = 77.80, *p* = .115).

3 Applying a Bonferroni correction within each hypothesis (i.e., the predicted effect of training on (a) new negative words and (b) negative intrusions) results in a corrected alpha of .0125 for these analyses, leaving the pattern of findings qualitatively unchanged.
